# Death-associated protein 3 in cancer—discrepant roles of DAP3 in tumours and molecular mechanisms

**DOI:** 10.3389/fonc.2023.1323751

**Published:** 2024-01-30

**Authors:** Hao Song, Huifang Liu, Xiufeng Wang, Yuteng Yang, Xiangkun Zhao, Wen G. Jiang, Laijian Sui, Xicheng Song

**Affiliations:** ^1^ The Second Medical College, Binzhou Medical University, Yantai, China; ^2^ Department of Otorhinolaryngology, Head and Neck Surgery, Yantai Yuhuangding Hospital, Qingdao University, Yantai, China; ^3^ Department of Nursing, Zhaoyuan People's Hospital, Yantai, China; ^4^ Cardiff China Medical Research Collaborative, Division of Cancer and Genetics, Cardiff University School of Medicine, Cardiff, United Kingdom; ^5^ Department of Orthopedics, Yantai Yuhuangding Hospital of Qingdao University, Yantai, China

**Keywords:** DAP3, cancer, apoptosis, molecular signaling, tumor progression

## Abstract

Cancer, ranks as the secondary cause of death, is a group of diseases that are characterized by uncontrolled tumor growth and distant metastasis, leading to increased mortality year-on-year. To date, targeted therapy to intercept the aberrant proliferation and invasion is crucial for clinical anticancer treatment, however, mutant expression of target genes often leads to drug resistance. Therefore, it is essential to identify more molecules that can be targeted to facilitate combined therapy. Previous studies showed that death associated protein 3 (DAP3) exerts a pivotal role in regulating apoptosis signaling of tumors, meanwhile, aberrant DAP3 expression is associated with the tumorigenesis and disease progression of various cancers. This review provides an overview of the molecule structure of DAP3 and the discrepant roles played by DAP3 in various types of tumors. Considering the molecular mechanism of DAP3-regulated cancer development, new potential treatment strategies might be developed in the future.

## 1 Introduction

Cancer remains a major threat to life expectancy in the 21st century, leading to a heavy burden on patients’ families, as well as the social healthcare system (1). Although the average life expectancy has further increased, increasing numbers of the aging population are struggling with cancer. Currently, a panel of diagnostic and therapeutic methods aimed at an early screening and effective treatment are applied for cancer treatment such as surgery, radiation therapy, and chemotherapy manifestied systemic therapy to improve cancer prognosis (2). In recent years, with the development of intensive clinical research, new therapies have emerged, in which molecular targeted therapy and immunotherapy present promising prospects (3).

Death associated proteins, DAPs, are a small group of proteins mediating interferon gamma-induced programmed cell death (4, 5). Initially, *DAPs were* identified as a group of novel genes encoding multiple biologically active proteins to induce cell apoptosis and anoikis (6). The encoded proteins include a proline rich cytoplasmic protein (DAP1), a novel calcium/calmodulin regulated kinase (DAP2/DAP-kinase DAPK) with anchor protein repeats, a death domains nucleotide binding protein (DAP3), and a new homolog of eIF4G translation initiation factor (DAP5) (7). DAP2 is a Ca^2+^/calmodulin-regulated enzyme that can induce apoptosis and autophagy (8). DAP5, an eIF4G family member and a mediator of cap-independent translation, impacts cell survival during mitosis (9). DAP1 has been shown to modulate autophagy (10) and presents low expressed levels in several malignancies, including breast cancer (11), neurological tumors (12), and pancreatic cancer (13). Previous studies have reported that methylation of the *DAPK* gene promoter and gene silencing might be associated with tumorigenesis, dissemination, and prognosis (14–17). In recent years, DAP3 has been found to be closely related to both tumor progression (18) and the resistance of tumor cells to chemotherapy (19–22). Further study will shed light on the mechanism of DAP3-mediated drug resistance and tumor metastases, providing new strategies for the targeted treatment of tumors.

## 2 Structure of *Dap3* and its cellular function

**Figure 1:**
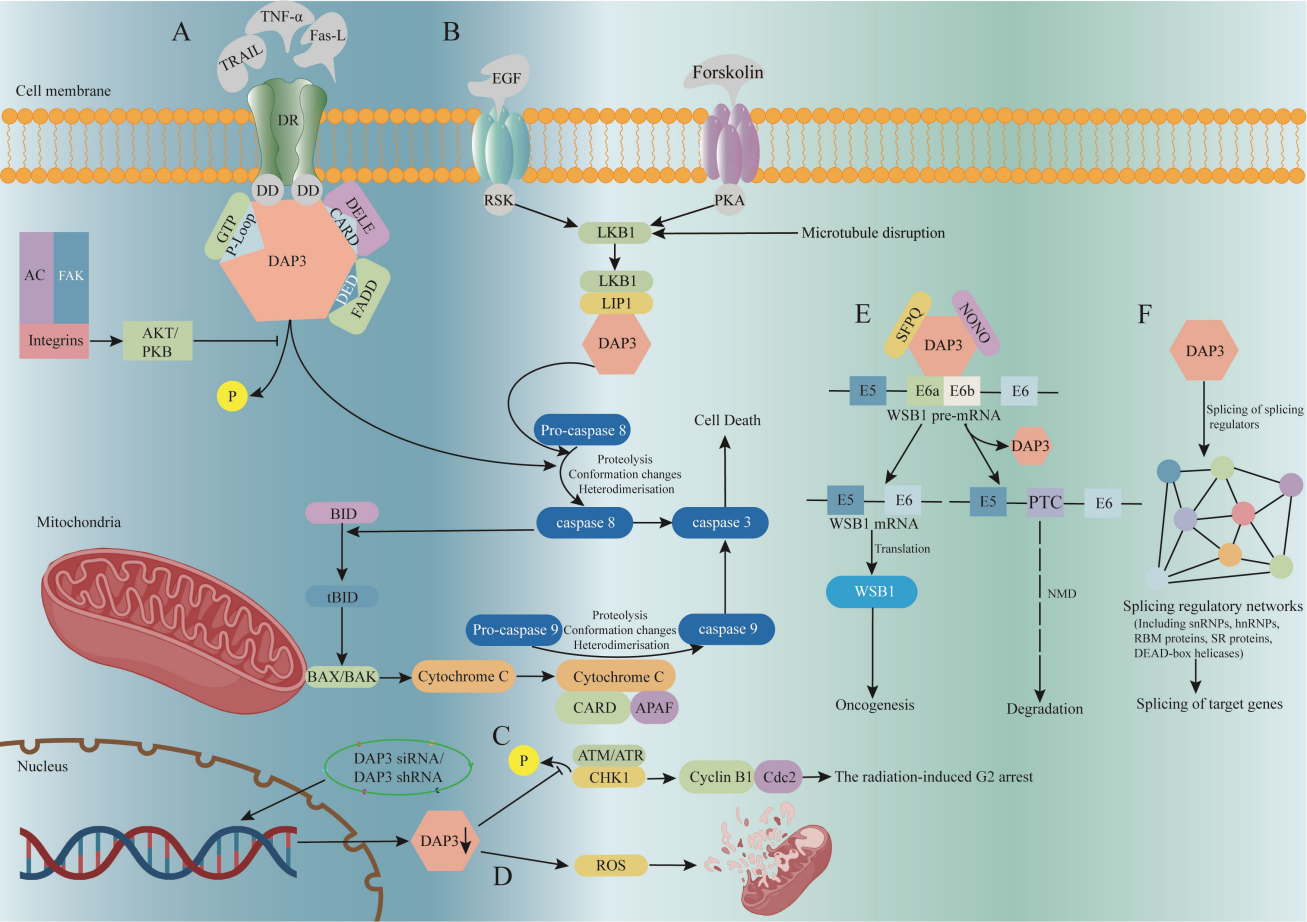
The signaling pathway and function of DAP3. **(A)** DAP3 is involved in the TRAIL, Fas-L, and TNF-α-mediated apoptosis pathways. **(B)** Co-expression of LKB1 and DAP3 enhances TRAIL-induced apoptosis. **(C)** DAP3 downregulation reduces p-CHK1 expression, inhibiting cell cycle G2/M blockade. **(D)** Down-regulating DAP3 reduces mitochondrial network disruption and intracellular ROS production. **(E)** DAP3 directly regulates substrate-specific splicing changes by mediating the formation of ribonucleoprotein complexes. **(F)** By regulating the splicing of several splicing factors, DAP3 has an indirect effect on splicing. AC, actin cytoskeleton; AKT/PKB, protein kinase B; APAF, apoptotic protease activating factor; ATM, ataxia telangiectasia-mutated; ATR, ataxia telangiectasia and rad3-related; BAX/BAK, BCL2 associated X protein; BID, BH3 interacting domain death agonist; CARD, caspase activation and recruitment domain; Cdc2, cell division control protein 2; CHK1, checkpoint kinase 1; DAP3, death-associated protein 3; DD, death domains; DED, death effector domain; DELE, death ligand signal enhancer; DR, death receptor; DISC, death inducing signaling complex; FADD, fas associated death; LIP1, LKB1 interacting protein 1; LKB1, liver kinase B1; NMD, nonsense-mediated decay; NONO, Non-POU domain containing octamer binding; PKA, protein kinase A; ROS, reactive oxygen species; SFPQ, splicing factor proline and glutamine rich; tBID, truncated BH3 interacting domain death agonist; TNF, tumor necrosis factor; TRAIL, recombinant tumor necrosis factor related apoptosis inducing ligand;.

DAP3, also known as S29mt, MRPS29, and bMRP-10, was first reported by Kissil et al. 25 years ago as an apoptosis associated protein (5). The *DAP3* mRNA is 1.7 kb long and the gene is located on chromosome lq21 (23). The molecular weight of the encoded DAP3 protein is 46 kDa, including a functional region, the P-Loop, which can bind to the ATP/GTP. DAP3 is a component of the small subunit of the mitochondrial ribosome and is located at the lower part of the subunit, far from the substrate binding site. Electron microscopy revealed no counterpart of DAP3 in bacterial or cytoplasmic ribosomes (24–26). DAP3 is distributed in the mitochondrial matrix rather than being released into the cytoplasm during apoptosis (27, 28). Generally, the DAP3 content varies depending on the type of cell (29). As an essential gene in mammals, silencing of *DAP3* induced embryonic atrophy and serious stagnation of embryonic development. Defective mitochondrial morphology, including shrinking and swelling, was found in these DAP3-deficient embryos, whereas other organelles seem to be integrated (30). Previous research has shown that functional DAP3 is actively involved in tumor necrosis factor (TNF), Fas ligand (Fas-L), and TNF-related apoptosis inducing ligand (TRAIL)-induced apoptosis (5, 31, 32). When protein kinase B (AKT or PKB) is disrupted, dephosphorylated DAP3 binds to the FAS-associated death domain (FADD) through its death effector domain (DED) region to trigger the activation of caspase 8. Caspase8 is capable of degrading BH3 interacting domain death agonist (BID) to create the truncated BH3 interacting domain death agonist (tBID), which facilitates the formation of BCL2 associated X protein (BAX/BAK) oligomers in the mitochondrial membrane, leading to the release of cytochrome C into the cytoplasm and subsequent activation of Caspase 9, a well-known molecule that induces cell death (**Figure 1A**).

Anoikis was described as apoptosis resulting from the detachment of epithelial cells from the surrounding intercellular matrix and surrounding cells (33). Some researchers have reported that the FAK-caspase 8 axis is the center of anoikis signaling (34–36). Activation of caspase 8 in the context of anoikis is associated with the interaction of FADD of FAS-associated DISC, therefore, DAP3 is thought to be a critical molecule for anoikis. Upon silencing AKT/PKB activity, DAP3 is dephosphorylated and interacts with FADD, which promotes pro-caspase 8 to caspase 8 (37, 38). The FADD-DAP3 interaction requires the involvement of interferon-β promoter stimulator 1 (IPS1), which is a caspase activation and recruitment domain (CARD) bearing protein anchored on the mitochondrial outer membrane. IPS1 localizes the reaction to the mitochondrial membrane and recruits pro-caspase 8, thus triggering the caspase cascade (39). Human endogenous retroviruses (HERVs) was also reported to modulate the host gene expression via generating RNAs, in which, HERV-K (HML-10) was identified to negatively regulate the transcripts of DAP3, thereby inhibiting its pro-apoptotic role, whilst inactivation of HML-10 by antisense oligonucleotides (ASOs)significantly upregulates the DAP3 transcripts and efficiently facilitate apoptosis (40).

According to Kissil’s study (31), when a mutation from lysine 143 to alanine was introduced into DAP3, the nucleotide binding motif of the DAP3 P-Loop was altered, which attenuated DAP3’s pro-apoptosis regulatory role. In addition, the cytoplasmic activity of DAP3 is regulated by AKT/PKB-mediated phosphorylation, which inhibits the TNF family death receptor signaling-induced pro-apoptotic effect (37). Therefore, the role of DAP3 in cell apoptosis might be regulated by multiple factors, including gene expression, protein localization, and protein activation, which indicates that the specific mechanism of DAP3 in cell apoptosis is complex, yet remains unclear.

## 3 DAP3 and tumors

Previous analysis of DAP3 expression in a clinical cohort indicated that DAP3 was highly expressed in pancreatic tumor tissues and was significantly associated with shorter survival (13). However, *DAP3* silencing in breast cancer cells led to enhanced tumor progression, including increased adhesion, migration, and invasion (41). The diverse and sometimes contrasting roles of DAP3 in different cells and different tumor types are summarized in this review (**Table 1**).

**Table 1:** DAP3-interacting proteins and potential functions.

Interacting/Regulatory partners	Cancer type	DAP3 Expression	Function/Bio significances	Reference(s)
AKT/PKB	-	-	DAP3 is phosphorylated by kinase AKT (PKB), and active AKT can nullify apoptosis induction by DAP3.	(32)
LKB1**、**LIP1	OS	–	LIP1 binds to LKB1 and anchors LKB1 to cytoplasm. Endogenous DAP3 could interact with LKB1 in osteosarcoma cells. Expression of LKB1 induced apoptosis and co-expression of LKB1 with DAP3 strongly induced apoptosis in osteosarcoma cells.	(45, 51)
Integrin	GBM	↑	Integrin-activation increases the level of Dap-3 to sustain migration.	(54)
-	THCA	↑	When thyroid tumors have a rich mitochondrial content, whether they belong to the oxyphilic tumor categories, to the papillary carcinomas or UMP type, DAP3 overexpression is dependent on the cell mitochondrial content.	(56, 58)
-	THYM	↑	The DAP3 mRNA level was positively correlated with the stage of the disease defined in the World Health Organization (WHO) classification.	(18)
CHK1	LUNG	↑	Downregulated DAP3 reduces p-CHK1 expression to inhibit cell cycle G2/M blockade.	(63, 67–71)
LGR5	STAD	↓	Downstream LGR5 expression was upregulated in DAP3-deficient stomach cancer cells, while down-regulation of LGR5 re-sensitized DAP3-deficient stomach cancer cells to 5-FU and oxaliplatin.	(20–22)
HSP90	BRCA	↓	Silencing of DAP3 leaded to the promoted tumor progression including the enhanced adhesion, migration and invasion in breast cancer cells. HSP90 and DAP3 expression patterns in breast cancer are comparable. Breast cancer metastasis and local recurrence are linked to decreased expression of HSP90.	(87, 88)
-	PAAD	↑	Levels of DAP3 transcripts in pancreatic cancer tissues were elevated compared to those in normal tissues Patients with high levels of DAP3 had a significantly shorter overall survival than those with low levels.	(13)
DELE1	COAD	↑	The tumor staging of COAD was correlated with DAP3 expression level. High DAP3 expression was associated with the poorer OS, DFS, DMFS and RFS according to the analysis in the clinical cohort. DELE1 is considered to be involved in coordinating the cell death process by interacting with DAP3.	(94)
ROS	-	-	Down-regulating DAP3 reduces mitochondrial network disruption and intracellular ROS production.	(27)
GR	-	-	GR binds to the P-loop of DAP3, in addition to helix-loop-helix/Per-Arnt-Sim proteins, HSP90 and other nuclear receptors to form the GR-HSP90 complex, which is involved in glucocorticoid receptor-induced apoptosis	(111, 112)
Drp1	-	-	The deletion of DAP3 significantly decreased the phosphorylation of Drp1 at mitochondrial Ser-637 and increased the residence time of Drp1 puncta on mitochondria during fission.	(62)
ADAR	-	-	DAP3 interacts with ADAR proteins and inhibits A-to-I RNA editing.	(124)

OS, Osteosarcoma; GBM, Glioblastoma; THCA, Thyroid Cancer; THYM, Thymoma; LUNG, Lung Cancer; STAD, Stomach Cancer; BRCA, Breast Cancer; PAAD, Pancreatic Cancer; COAD, Colorectal Cancer.

### 3.1 DAP3 and osteosarcoma

Osteosarcoma is the most common primary malignant tumor of the skeleton. Despite recent improvements in chemotherapy and surgical treatment, it is still difficult to obtain a satisfactory prognosis for osteosarcoma (42). As a member of the TNF family (43, 44), TRAIL is considered as a selective apoptosis inducer in most tumors (45, 46), rendering it a potential target for tumor therapy. DAP3 has been revealed to play a vital role in TRAIL-mediated apoptosis through the activation of pro-caspase 8 (32). Liver kinase B1 (LKB1) was proposed as a tumor suppressor and cell cycle regulator, which was initially discovered as the mutant gene in Peutz-Jeghers syndrome (PJS) (47–49). Recently, aberrant LKB1 expression was also found to be associated with the progression and abnormal cell cycles of tumors (50–52). While screening molecules binding to DAP3 in cDNA libraries using the yeast two-hybrid method, Takedade et al. found that LKB1 associated with DAP3 in osteosarcoma cells, mediated by LKB1 interacting protein 1 (LIP1) (53). Co-expression of LKB1 and DAP3 was reported to enhance TRAIL-induced cell apoptosis (53), whereas DAP3-induced apoptosis was reduced when LKB1 was mutated. Therefore, LKB1 and DAP3 are thought to be promising targets for osteosarcoma therapy (**Figure 1B**).

### 3.2 DAP3 and glioblastoma

Glioma, also known as neuroglioma, is one of the most common tumors of the nervous system. Glioma is characterized by high susceptibility to recurrence, which often induces severely damaged cognitive function, despite the lesion being surgically removed, followed by systemic therapy. Research has found that the migration rate of glioma *in vitro* is closely related to its aggressiveness *in vivo* (54). To explore the genetic determinants of glioma invasion, Mariani (55) compared the gene expression profiles of glioblastomas in the tumor center and invasive margins using quantitative real-time reverse transcription PCR (RT-qPCR) and found that *DAP3* was overexpressed in invasive glioma cells. Meanwhile, silencing of *DAP3* in glioblastoma cells attenuated migration. When the glioma cell line, T98G, was placed on laminin and extracellular matrix (ECM), both the mRNA expression and protein levels of DAP3 were upregulated and the cell resistance to apoptosis was enhanced (55). The authors hypothesized that integrin activation increases *DAP3* transcript levels to enhance glioma cell migration, and secondary activation might alter the function of DAP3, reducing its pro-apoptotic activity. In addition, subsequent upregulation of DAP3 might cause the resistance of glioma cells to radiotherapy and chemotherapy (56).

### 3.3 DAP3 and thyroid cancer

Thyroid cancer is the most common endocrine malignant tumor with increasing incidence worldwide in the past three decades (56). DAP3 mRNA and protein expression were increased in thyroid tumors with mitochondrial biogenesis compared with that in the normal adjacent tissues, and upregulated cell growth-associated proteins ETS transcription factor ELK1 and estrogen related receptor alpha (ESRRA) were also associated with DAP3 overexpression in thyroid tumors (57). The expression of DAP3 also depends on the number of mitochondria in aerobic thyroid tumors, papillary thyroid carcinoma, and oncological potential undefined thyroid carcinoma (58). In thyroid oncocytoma, upregulated DAP3 expression was reported to be closely associated with attenuated apoptosis (57). The transcription of *ELK1* plays a role in the promotion of early genes, such as c-fos, an *in situ* oncogene (59), whereas the transcription factor binding site sequence of ESRRA has specificity for the small mitoribosomal subunit. These results, together with the participation of DAP3 in the composition of mitochondrial ribosome small subunit, indicate that DAP3 might act as a regulator of mitochondrial protein synthesis to maintain mitochondrial homeostasis and is involved in the tumorigenesis of thyroid cancers.

### 3.4 DAP3 and lung carcinoma

Zhou (60) used the expression profile of lung adenocarcinoma in The Cancer Genome Atlas (TCGA) and constructed a gene interaction network using weighted gene co-expression network analysis to identify dozens of novel genes of opposite relevance, including the long noncoding RNA ATP13A4-AS1, and those encoding, HIG1 hypoxia inducible domain family member 1B, DAP3, and interferon stimulated exonuclease gene 20kDa-like 2 (ISG20L2). In addition, the team examined the expression levels of DAP3 in both tissues and cell lines of human lung cancer and carried out a functional analysis to determine its biological role, which showed that DAP3 was significantly elevated in lung cancer tissues and cells. *DAP3* knockdown in human lung cancer cell lines A549 and H1299 resulted in significantly reduced cell survival after radiotherapy. Mitochondrial respiration is a key process for cellular activity. In lung adenocarcinoma, several components of the chromatin reconfiguration complex were shown to have common mutations, resulting in increased oxidative phosphorylation and enhanced sensitivity to oxidative phosphorylation inhibitors. As a constituent of mitochondrial ribosomes, DAP3 plays a crucial role in the biosynthesis of proteins associated with the mitochondrial respiratory chain (61). Prior studies have indicated that the downregulation of DAP3 hampers the synthesis of these particular proteins (62). Hence, Sato et al. postulated that the impact of DAP3 on the synthesis of mitochondrial respiratory chain proteins could potentially influence the proliferation of A549 and H1299 cells (63). The authors demonstrated that low expression of DAP3 was associated with a good prognosis, emphasizing its potential value in the diagnosis and treatment of lung cancer.

Radiotherapy, chemotherapy, and surgery are routine methods for lung cancer treatment; however, the efficiency of radiotherapy might be attenuated by radiation resistance; therefore, it is vital to determine the molecular mechanisms of radiation resistance in lung cancer. Cell cycle checkpoints are ideal targets for sensitizing cancer cells to radiotherapy. Studies showed that some human cancer cells can be sensitized to radiotherapy by eliminating G2 resistance (64–66). Checkpoint kinase 1 (CHK1) can be reactivated by radiotherapy, leading to cell cycle G2/M block via inactivation of the cyclin B1 and Cdc2 complexes, while CHK2 activation by p53 leads to cell cycle G1 block (67, 68). It was discovered that some chemo- and radiation resistance involved molecules that were capable of regulating cell cycle blockade (69, 70). For example, *DAP3* knockdown in a lung carcinoma cell line reduced the expression of radiotherapy-induced phosphorylated CHK1, which in turn led to radiotherapy-induced G2 arrest (63). In addition, since ataxia telangiectasia mutated (ATM), ATP, and ataxia telangiectasia and Rad3-related protein (ATR) are all involved in the regulation of CHK1 and CHK2 phosphorylation, it is possible that DAP3 regulates radiotherapy-induced p-CHK1 by modulating ATM, ATP, or ATR (**Figure 1C**) (71).

Recent studies have shown that retinoic acid-inducible gene-I (RIG-I)-like receptors (RLRs) activation presents anti-tumor effects, including anti-tumor immunity and cell death (72–74). Sato (75) previously reported that the RLR agonist [Poly(I:C)] enhanced radiosensitivity, and that cotreatment with Poly(I:C) and ionizing radiation (IR) more than additively increased cell death in lung adenocarcinoma cells, indicating that Poly(I:C) modulates the cellular radiation response. Sato (75) found that Poly(I:C) inhibited the translation of *DAP3* mRNA and decreased the DAP3 protein level, which increased IR-induced cell death. These results highlight the importance of DAP3 in the cellular radiation response of human lung adenocarcinoma cells and improve our understanding of DAP3-mediated radioresistance mechanisms, with implications for the efficacy of radiation therapy for lung adenocarcinoma.

### 3.5 DAP3 and gastric carcinoma

As one of the most prevalent malignant gastrointestinal tumors, gastric cancer accounts for the third-highest death rate worldwide (76). DAP3 expression was not detected in wild-type human gastric cancer cell lines, BGC-823 and HGC-27. DAP3 exerts a pivotal role in cell apoptosis, an important mechanism regulating the proliferation of malignant tumors; therefore, it was suggested that DAP3 deficiency might be related to the progression of gastric cancer. When gastric carcinoma cells were treated with certain concentrations of recombinant human tumor necrosis factor alpha (rhTNF-α) and 5-fluorouracil (5-FU), the proliferation of both BGC-823 and HGC-27 cells was suppressed; meanwhile, DAP3 expression was detected in the treated cell lines (77). Therefore, rhTNF-α and 5-FU were proposed to bring about a DAP3-induced apoptotic process. When the cell death receptor binds to rhTNF, which further activates the death region (DD), DAP3 connects the DD to the responsive protein FADD to activate caspase 8. The binding site in DAP3 is the structural domain with an apoptosis-positive effect containing the P-loop at the carboxyl terminus, forming a death-inducing signaling complex, which in turn activates caspase 8 to induce apoptosis (32).

Another study on gastric cancer also found higher levels of DAP3 expression in highly or moderately differentiated tumors by RT-PCR and immunohistochemistry (19). According to T-staging and TNM staging, the expression level of DAP3 showed a decreasing trend in more advanced tumors, which indicated that DAP3 expression correlated significantly and negatively with the prognosis of gastric cancer. By contrast, an *in vitro* migration assay showed that gastric cancer cells with DAP3 downregulation presented a more invasive phenotype, which was consistent with the results of DAP3 expression analysis in a clinical gastric cancer cohort: lower DAP3 expression in patients who presented higher local recurrence and/or distant metastases (19). To explore the potential mechanism of DAP3 in chemotherapy resistance, investigators induced apoptosis in gastric cancer AGS and HGC27 cells using 5-FU and oxaliplatin. However, the apoptosis induced by chemotherapeutic drugs could be interrupted by *DAP3* silencing. Therefore, it was hypothesized that low DAP3 expression might reduce sensitivity to 5-FU and oxaliplatin in gastric cancer and DAP3 was proposed as a potential molecular marker to predict the efficiency and prognosis of preoperative chemotherapy in patients treated with combined chemotherapy for gastric carcinoma (19).

Moreover, DNA damage-induced apoptosis mainly functions through the mitochondrial pathway (78–80), suggesting that DAP3 might impact the efficacy of chemotherapy-induced apoptosis through the mitochondrial pathway. Recently, Jia and his coworkers (20) determined the expression levels of Leucine-rich G-protein coupled receptor 5 (LGR5), an important downstream molecule of β-catenin signaling (21, 22), in gastric cancer cell lines (MGC803 and HGC27) with low DAP3 expression and found that LGR5 expression was upregulated in these DAP3-deficient gastric cancer cells, while downregulation of LGR5 re-sensitized DAP3-deficient gastric cancer cells to 5-FU and oxaliplatin. The Wnt/β-catenin signaling pathway is closely associated with drug resistance in tumors (81, 82), and inhibition of this pathway contributes to improved chemo-resistance (83), making the β-catenin pathway a promising therapeutic target to treat chemo-resistance. LGR5 was demonstrated as an important downstream target of β-catenin signaling (21, 22), and aberrant LGR5 expression reduced apoptosis in gastric cancer cells treated with 5-FU and oxaliplatin by suppressing caspase 3 cleavage. Therefore, it was speculated that targeting LGR5 could be a promising therapeutic strategy to improve chemoresistance in DAP3-deficient gastric cancer cells.

### 3.6 DAP3 and breast cancer

As the most common invasive tumor, breast cancer often occurs in women over 30 years of age (84, 85). Levels of DAP3 were low in breast cancer (18, 86) compared with the respective normal counterpart tissues and silencing of *DAP3* promoted tumor progression, including enhanced adhesion, migration, and invasion in breast cancer cells (87), which indicated that increased expression of *DAP3* was a favorable marker of prognosis in human breast cancer. In a clinical cohort study, significant correlations between DAP1 and DAP3 were found in breast cancer (87), another study reported that the expression patterns of DAP3 and heat shock protein 90 (HSP90) were similar. HSP90 was reported to correlate negatively with metastasis and local recurrence of breast cancer (88). Furthermore, subgroup analysis showed a substantial impact of DAP3 on the prognosis of breast cancer subtypes, except for basal cell-like subtypes, estrogen receptor-negative subtypes, and human epidermal growth factor receptor-2 overexpression subtypes (P < 0.05). Bruceine D (BD) is a quassinoid isolated from the traditional Chinese herbal medicine made from the fruit of *Brucea javanica*, which exhibits anti-cancer effects (89, 90). Further analysis using the Gene Expression Omnibus database showed that BD could reduce the expression of DAP3 in the luminal A subtype of breast cancer (MCF7 cells), indicating that DAP3 might be a possible target for BD intervention in breast cancer (91). Wang et al. found that BD could inhibit the energy metabolism and proliferation of breast cancer cells (MDA-MB-231) through the phosphatidylinositol-4,5-bisphosphate 3-kinase (PI3K)/AKT signaling pathway. Upon loss of AKT activity, DAP3 dephosphorylates and interacts with FADD and induces caspase 8 production (37). Therefore, it was speculated that BD could downregulate the expression of DAP3 through the PI3K/AKT signaling pathway, thus inhibiting the proliferation of breast cancer.

### 3.7 DAP3 and pancreatic cancer

Pancreatic cancer remains one of the most fatal malignancies worldwide, accounting for 466,000 cancer deaths in 2020. The number of patients who died from the disease almost equaled the cases diagnosed (496,000) (92). In contrast to the advances and improvement in the diagnosis and treatment of other malignancies, the demand for both early detection and effective therapeutic approaches for pancreatic cancer requires a better understanding of corresponding molecular and cellular machinery. A study using a large cohort of patients with pancreatic cancer together with a small public database (13), reported the clinical and survival benefits of DAP3 in patients with pancreatic cancer. When compared with that in normal tissues, cancer tissues from 223 patients with pancreatic cancer expressed higher levels of DAP3, which did not correlate with DAP1 expression levels. However, there was no discernible difference in the levels of DAP3 expressed by tumor tissues from various anatomical sites. Levels of *DAP3* transcripts in pancreatic cancer tissues were elevated compared with those in normal tissues. Patients with high levels of DAP3 had a significantly shorter overall survival than those with low levels (p = 0.012). Additionally, the effect of DAP3 status and lymph node status on patient survival was cross-analyzed. Patients with high DAP3 and lymph node-positive tumors had the worst prognosis. The combination of DAP3 expression and nodal status significantly improves its efficacy as an independent survival predictor (13).

### 3.8 DAP3 and colorectal cancer

As a major type of cancer worldwide, colon cancer can be surgically removed if the tumor is diagnosed early, although chemotherapy and radiotherapy are essential for patients with advanced stage disease (93). However, because of severe side effects and low overall survival rates after chemotherapy, it is essential to reveal the molecular mechanism of colon cancer to develop new therapeutic strategies. Research by Sui and his coworkers demonstrated that the expression of DAP3 was increased in colon cancer tissue compared with that in normal adjacent tissue at the mRNA and protein level (94). DAP3 mRNA and protein expression also correlated with the tumor staging (94). High DAP3 expression was associated with poorer survival according to analysis in the clinical cohort. To determine the role of DAP3 in colon cells, *in vitro DAP3* knockdown cell models were created using Ribozyme. The results of cell toxicity tests showed that downregulated DAP3 expression increased cell sensitivity to the chemotherapeutic drugs in RKO cells (94). In the same study, differential DAP3 expression was also observed in patients with different Bevacizumab responses (94). Bevacizumab functions as an inhibitor of tumor angiogenesis; therefore, a panel of biomarkers was applied to investigate the correlation between *DAP3* and neovascularization in the TCGA-Colon Adenocarcinoma dataset (94). From the analysis, *DAP3* expression was inversely correlated with most of the angiogenesis biomarkers, such as vascular endothelial growth factor C (VEGFC), angiopoietin 2 (ANGPT2), platelet and endothelial cell adhesion molecule 1 (PECAM1), sphingosine-1-phosphate receptor 1 (S1PR1), and S1PR2, which indicated that high *DAP3* expression might actively suppress the angiogenesis in colon tumors (94). Notably, DAP3 binding cell death enhancer 1 (DELE1), also known as KIAA0141, was identified as the mitochondrial protein cleaved by the Metallo endopeptidase OMA1 (overlapping activity with M-AAA protease). Full-length DELE1 was cleaved into a shorter fragment (S-DELE1) in the cytosol to transmit the mitochondria stress signals, and the stress was activated through a heme-regulated inhibitor (HRI)-dependent pathway, which relayed the mitochondrial stress to activating transcription factor 4 (ATF4) (94). It was suggested that DELE1, via the OMA1-DELE1-HRI mitochondrial pathway, might mediate both detrimental and beneficial responses, depending on the mitochondria stress sources (95). DELE1 was reported to act as the upstream molecule that activates CASP3, CASP8, and CASP9 to induce cell apoptosis. *DELE1* silencing suppressed caspase activation and enhanced viability (96). Silencing *DELE1* also reduced death receptor (DR)-mediated apoptosis; therefore, DELE1 is considered to be involved in coordinating the cell death process by interacting with DAP3. The DELE1 protein contains a mitochondrial targeting sequence at its N-terminus and two tetrapeptide repeats in the protein-protein interaction domain, which is important in the mitochondrial stress signaling pathway (97, 98). *DAP3* knockdown led to mitochondrial fragmentation (62, 75). These findings suggested that DAP3 regulates cell function and cell death through the mitochondrial signaling pathway by interacting with DELE1. In Sui’s report, significantly reduced *DELE1* mRNA expression was found after *DAP3* knockdown in RKO cells. Knockdown of *DAP3* and *DELE1*, respectively, in colon cancer CRC cell lines sensitized the cells to 5-FU, Methotrexate, and Docetaxel, and cells with simultaneous knockdown of *DAP3* and *DELE1* were markedly sensitive to all the drugs tested, compared with the single knockdown lines and the controls. DELE1 was subsequently found to be a key component, together with OMA1 and HRI, in a mitochondria stress signaling pathway (95, 97, 98), thus DAP3 and DELE1 might lead to mitochondria-associated drug resistance (99, 100). However, the exact links between mitochondrial function and drug resistance induced by DAP3 are less clear and require further investigation.

### 3.9 DAP3 and liver cancer

Primary liver cancer is one of the six most common types of cancer and is the third leading cause of cancer deaths worldwide (92). Despite the existence of a wide array of treatment options, the rates of recurrence and mortality for liver cancer remain concerning. Resistance to apoptosis is a crucial characteristic of numerous cancer cells, which promotes their survival (101). In an effort to enhance the accuracy of liver cancer prognosis and promote treatment personalization, Wang (102) introduced a novel feature model to predict apoptosis-related prognosis in HCC, which comprises nine genes, including *DAP3*. This model was developed using TCGA expression data and a list of 161 apoptosis-related genes from gene set enrichment analysis. Based on median risk scores, the researchers divided the patients into high- and low-risk groups. The findings indicated that the survival rate of patients with low risk scores was significantly superior to that of patients with high risk scores and the high-risk group was significantly more in immune cell infiltration and with higher immunoscore and stromalscore than in the low-risk group (102). Chen (103) employed weighted gene co-expression network analysis to identify genes associated with HCC (*BAK1* (encoding BCL2 antagonist/killer 1), *SPP1* (encoding secreted phosphoprotein 1), *BSG* [encoding basigin (Ok blood group)], *PBK* (encoding PDZ binding kinase), and *DAP3*) and establish a predictive risk model. In that study, the International Cancer Genome Consortium and Gene Expression Omnibus datasets were employed to validate the performance of models associated with apoptosis-related prognostic risks. The findings indicated that patients with high risk scores had a reduced chance of survival and were more prone to die as compared to those with low risk scores. The expression levels of prognostic genes were significantly upregulated in the high risk group compared with those in the low risk group, except for *DAP3*. By analyzing the correlation between infiltrating immune prognostic and apoptosis-related genes with risk scores, the investigators discovered that risk scores correlated significantly and positively or negatively with the majority of immune cells. Furthermore, prognosis-related genes were found to be highly correlated with the majority of immune cells, with the exception of *DAP3*. To investigate the potential mechanisms of DAP3 in HCC, Zhang et al. conducted a KEGG pathway analysis using differentially expressed genes between subgroups with varying DAP3 expression levels. The analysis revealed that several pathways related to cell proliferation and metabolism were significantly activated in the group with high DAP3 expression (104), which indicates the oncogenic role of DAP3 in HCC. In addition, during the analysis, “cell cycle,” “DNA replication” and “Wnt signaling pathways” were significantly enriched when DAP3 was upregulated. GSEA demonstrated that “G2M checkpoint,” “Mitotic spindle,” Myc targets,” “DNA repair” and “E2F targets,” which are related to cancer development and progression, were significantly activated in patients with high DAP3 expression (104). In addition, recent studies have shown that the Wnt pathway is involved in regulating immune infiltration in the tumor microenvironment (105–108). Results based on the TCGA database showed that innate immune cells, including neutrophils, dendritic cells, and natural killer cells, were negatively correlated with DAP3 expression (104). In patients with high DAP3 expression, the infiltration of adaptive immune cells responsible for anti-tumor responses, such as B cells, T cells, CD8+ T cells, and cytotoxic cells, was significantly inhibited. It is suggested that the expression of DAP3 may be related to the immunosuppressive tumor microenvironment of HCC (104). These results indicated that DAP3 may serve as an oncogene in HCC.

Abnormally expressed circRNA is associated with the progression of HCC. Silencing hsa_circ_0002003 can inhibit the proliferation, migration, and invasion abilities of HCC Huh7 cells and down-regulate DAP3 expression (109). On the contrary, the proliferation and mobility of MHCC97H cells were significantly enhanced after overexpression of hsa_circ_0002003 (109). In conclusion, hsa_circ_0002003 may play critical roles in HCC pathogenesis and may serve as a potential biomarker of HCC.

### 3.10 DAP3 and thymoma

Thymoma is one of the most common solid tumors in the mediastinum (110). To date, there have been few studies on DAP3 and thymic carcinoma. To identify cytological differences between non-invasive and invasive/metastatic thymomas, Sasaki et al. detected differentially expressed genes in 36 patients with invasive/non-invasive thymomas on chromosome 1q using a microarray and quantitative real-time reverse transcription PCR analysis. The results confirmed that the expression levels of Abelson-related gene protein (Arg) and DAP3 were significantly higher in invasive thymomas (stage IV thymomas) than in stage I thymomas (18). With the maturation of technology and further in-depth research, the involvement of DAP3 in the pathogenesis of thymic carcinoma will be revealed.

## 4 DAP3 regulates glucocorticoid receptors

A study demonstrated that DAP3 could increase steroid sensitivity and induce a ten-fold increase in transcriptional activation, which was triggered by glucocorticoid receptor ligands (111). Hulkko (112) then discovered that in addition to the helix-loop-helix/Per-Arnt-Sim protein, the glucocorticoid receptor (GR) binds to the P-loop of DAP3, heat shock protein 90 (HSP90), and other nuclear receptors, to form an aggregate. This finding suggested that DAP3 is involved in glucocorticoid receptor-induced apoptosis and that this mechanism might contribute to the formation of the GR-HSP90 complex in the cytoplasm.

## 5 DAP3 is involved in mitochondrial regulation

Mitochondria provide energy in the form of ATP for cell metabolism. Mitochondrial homeostasis is balanced by the opposing fusion and fission processes, which enable cells to adapt to changing physiological conditions (113, 114). Mitochondrial dynamics are modulated by a series of proteins such as dynamin-related protein 1 (Drp1) (115), the mitochondrial fusion protein 2 (Mitofusion2, Mfn2) (116), and optic atrophy-associated protein 1 (OPA1) (116).

DAP3 was reported to be a mitochondrial ribosomal component mainly localized in the matrix of mitochondria, which is pivotal to regulating mitochondrial function (62). In *Dap3* knockout mouse embryos, shrunken mitochondria and swollen cristae were observed (30). Moreover, significantly increased mitochondria fragmentation was observed in DAP3-depleted Hela cells. Upon DAP3 reintroduction, mitochondrial fragmentation was rescued, which suggested that DAP3 was essential to maintain the mitochondrial network (62). DAP3, as a mitochondrial ribosomal protein, was observed to regulate the synthesis of mitochondrial proteins, which subsequently modulated the physiological function of mitochondria, such as ATP production and the ΔΨm (62). Further research on the molecular mechanism revealed that DAP3-induced mitochondrial fragmentation was dependent on mitochondrial fission factor (Mff)-Drp1 fission activity, accomplished by mediating Drp1 phosphorylation at Ser-637 (62), which was critical to modulate mitochondrial dynamics (117).

Autophagy is a highly conserved process of self-degradation to maintain cell homeostasis (118). Mitochondria were reported to provide the membrane source to facilitate autophagy by introducing reactive oxygen species (ROS) (119) (**Figure 1D**). As an important mitochondrial matrix protein, the biological effects of DAP3 also affect autophagic activity. Under intrinsic stress, such as Earle’s Balanced Salt Solution (EBSS)-induced starvation, the expression levels of LC3-II, a marker of autophagy, were significantly reduced in DAP3-depleted cells, indicating that *DAP3* silencing sensitized cells to the intrinsic mitochondrial mediated death pathway (62). In addition, DAP3-mediated apoptosis was also reported to be induced via an extrinsic pathway (5, 32).

hNOA1, the human homologue of AtNOA1 (Arabidopsis thaliana nitric oxide-associated protein 1), was reported as a large GTP-binding protein that was located within mitochondria like DAP3. Besides the mitochondrial dynamics regulating role, hNOA1 was also found to directly interact with DAP3. Labeled DAP3 was especially pulled down by GST-hNOA1, rather than GST, from HEK293 cell lysates, which renders hNOA1 a putative interacting partner of DAP3. Interestingly, hNOA1 exerted a similar role as the regulator for interferon-γ induced apoptosis, silencing hNOA1 protected cells from interferon-γ mediated cell death as the DAP3 depleted cells. Study using siRNA to knockdown hNOA1 partially protected Hela cells from staurosporine-induced mitochondrial fragmentation characterized cell apoptosis (120). How DAP3 interacts with hNOA1 and how to orchestrate different mitochondrial functions still need to be further determined.

## 6 DAP3 facilitates the induction of substrate-specific splicing by the nuclear protein complex

RNA-binding proteins (RBPs) are involved in multiple aspects of RNA processing and regulation, including RNA transcription, splicing, cleavage, modification, degradation, transport, and translation. The multiple RBPs form a regulatory network that exerts complex and dynamic transcriptome and proteome control. The processing and regulation of RNA by RBPs are essential for the normal development and physiology of the individual; therefore, any disturbance of RNA processing might lead to disease (121). Aberrant RNA splicing is observed in almost all types of cancer (122), and selective splicing in cancer cells can convert genes from tumor suppressor or non-oncogenic subtypes to oncogenic subtypes. Han (123) identified DAP3 as a widespread splicing regulatory RBP involved in regulating several splicing events using enhanced UV cross-linking combined with immunoprecipitation sequencing, transcriptome sequencing, and proteomic analysis. In multiple cancer types, *DAP3* is a strong oncogenic protein that interacts with adenosine deaminase RNA specific (ADAR) proteins and inhibits A-to-I RNA editing (124). There are two different ways by which DAP3 carries out its splicing regulation functions. First, DAP3 mediates the recruitment of splicing factors, such as splicing factor proline and glutamine rich (SFPQ) and non-POU domain containing octamer binding (NONO), to the binding sites by first directly binding to the target RNA. Second, DAP3 fine-tunes the splicing pattern of hundreds of splicing components, which indirectly modifies splicing. Global splicing alterations were observed to play a role in carcinogenesis when DAP3 was overexpressed in several types of cancer (**Figures 1E, F**). WSB1 WD repeat and SOCS box-containing protein 1 (WSB1) is an e3-ubiquitin ligase that can promote ATM ubiquitination and degradation, leading to tumor progression (125). Functional studies of WSB1 non-productive splicing provide evidence for a causal relationship between DAP3’s regulatory functions and tumorigenesis, providing key mechanistic insights into the role of DAP3 in splicing regulation in cancer development.

## 7 Conclusion and Prospects

Tumorigenesis is a complex process with multiple steps involving various molecules. Determining the molecular mechanism of tumor occurrence will contribute significantly to the development of novel targeted cancer treatments. The current review revealed inconsistencies in the role played by DAP3 in cells. DAP3 is highly expressed in pancreatic cancer (13), glioblastoma multiforme (55), advanced stage thymomas (18); and in non-epithelial derived tumors, Burkitt Lymphoma, and in a subtype of acute lymphoblastic leukemia according to an earlier study (126). In contrast, the levels of DAP3 are low in gastric cancer (20) and breast cancer (86) compared with the adjacent normal tissues. Interestingly, DAP3 is an indicator of patients’ responses to drug and radiation therapies in cells derived from certain solid cancers, and *DAP3* knockdown markedly increased the rate of cell death and reduced the fraction of cell survival in response to radiation and treatment by chemicals (19, 81–83). In the human hepatoma cell line, Hep3B, *DAP3* is one of the prominent responsive genes regulated by a P53 regulating protein TP63 (127). In human breast cancer, DAP3 was found to interact with HSP90 (88). The reasons for the discrepancies in the role played by DAP3 in cells are unclear. Interestingly, aberrant DAP3 expression can facilitate tumor progression rather than promote apoptosis, although DAP3 was initially identified as a pro-apoptotic protein. The relationship between tumor promotion and cell apoptosis induced by DAP3 is still unclear.

Targeted therapeutic drugs are essential in tumor research, greatly minimizing the suffering of patients with confirmed tumors. The present review on the important role of DAP3 in tumorigenesis and progression strongly advocates a therapeutic role for targeting DAP3. According to previous studies, DAP3 is associated with tumor proliferation, metastasis, chemo-resistance, and radiotherapy resistance; however, the exact mechanism is unclear. Contrasting roles of DAP3 in the tumour have progressed significantly in the past decades, however, a panel of questions remains to be answered. For example, the signalling transduction of DAP3 is not well elucidated currently. How does DAP3 signalling interact with the other signallings such as Wnt, β-catenin, TRAIL signalling to present discrepant roles in cancers? Which is the dormant death agent for the mediated cell apoptosis process, the extrinsic pathway or the intrinsic one? The present report on the oncological role of DAP3 in tumourigenesis and progression strongly argues for a therapeutic role by targeting DAP3. Molecular signatures are yet to be found for these purposes. Research on DAP3 is currently underway, further investigation using advanced technology and multi-omics, the oncological role of DAP3 will eventually be elucidated, which will deepen our current understanding of the pathogenesis of tumour and provide evidence for developing novel therapies.

## Author contributions

HS: Writing – original draft. HL: Writing – original draft. XW: Writing – original draft. YY: Data curation, Writing – original draft. XZ: Data curation, Writing – original draft. WJ: Writing – review & editing. LS: Writing – review & editing. XS: Writing – review & editing.
